# Crowner’s Quest Law

**Published:** 1885-04

**Authors:** 


### CROWNER'S QUEST LAW.

The venerable farce, known as the coroner's inquest, is so frequently enacted in our community that it begins to excite interest as well as create amusement. Every new rendition of the piece shows that this ancient fossil of the law remains without change since the days of Hamlet. The setting of the piece in all the details, like the rules of the games of children, is handed down from generation to generation without change or improvement, and without the probability or hope of public benefit.

At the close of the performance the curtain falls, by the announcement of the verdict, (for which the jury is in no way responsible,) as unvarying as the other parts of the show.

In all cases of sudden death, except from external injuries, the cause is invariably found to be "congestion," or "heart disease." The hurried glance at the corpse, technically called "viewing the body," permits no better conclusion, and possibly accomplishes all the objects of the solemn nonsense, entitling the officers to legal fees and the body to Christian burial.

In the February number of this journal, allusion was made to one of these cases of fatal congestion, in which strong suspicions of poisoning were entertained, but the stereotyped verdict consigned both them and the body to the oblivion of the grave.

The following account of another case, in which the usual verdict was rendered, is copied, without comment, from the Atlanta *Constitution*:

NO. I. SCARED TO DEATH.

Lewis Perkins, a negro man whose home was at 89 Martin street, died suddenly yesterday morning. His death was a mystery to the people in that neighborhood, and Coroner Haynes decided to make a legal inquiry. Perkins was apparently a stout, healthy negro. For several months past he has been working for Walter A. Taylor. Saturday evening he quit work at the usual hour, and after drawing his pay went home. He ate a hearty supper and passed the evening talking to the family. When bedtime came he retired. He slept on a pallet on the floor in the same room with a colored man named Sutton. He slept soundly until about three o'clock when he awoke the other occupant of the room by giving utterance to the most piteous groans and calling loudly for help. Sutton sprang from his bed and hastened to the pallet while his wife made a light. The light showed that Perkins was lying flat upon his back with his eyes and mouth wide open. His features were greatly distorted. His eyeballs were nearly out of socket, and his general appearance indicated that he was badly frightened. Sutton placed his hand under Perkins' head, and raising him up asked:

"What's the matter, Lewis?"

Perkins made several attempts to speak. His jaws would move but his tongue failed to do his bidding. Finally, however, he appeared to concentrate his powers of speech, and with a terrible effort uttered the words:

"Cat, c-a-t, c-a—"

Before finishing the last word his eyes rolled about in his head, his body gave one immense shake, and he fell back dead. Early in life Perkins was severely bitten by a cat. His arm still shows the marks made by the vicious cat's ugly teeth. The bite made him averse to cats. He was actually afraid of them, and the several witnesses before the jury of inquest yesterday stated that they had seen Perkins run from a cat frequently. A cat was found in the room where Perkins died, and one witness asserted most positively that Perkins had been scared to death by a cat. Dr. Boring was unable to advise the jury until after making a post-mortem. The jury then returned a verdict of death from congestion of the lungs.

The account of another and more recent case of peculiar interest, copied from the same source, is subjoined. After observing the skill and professional acumen of the coroner in eliciting the evidence, and the poetic fervor of the clerk in reporting it, we are prepared for the sapient verdict.

A shade of doubt, however, remains upon the mind, whether the "dropsy of the heart" existed in the man or in the clock. Post-mortem examinations by skillful experts might have dispelled this doubt, and possibly have enriched science with an explanation of the mysterious nervous connection existing betwixt men, women and clocks, which has perplexed the learned ever since the birth of Tristram Shandy.

### No. 2.

Alexander J. Young, a well known railroad machinist, was found dead in his bed yesterday morning.

The discovery was made by Mr. Young's wife who occupied the same bed. Mr. Young has been living at 171 Houston street for some time past. The building is a two-story frame. The front portion of the first floor is used as a store room, while a room in the rear was occupied by Mr. and Mrs. Young as a bed room. The store has been managed by Mrs. Young, while her husband was away from home during the day working at his trade. The bed-room was simply and neatly furnished, and in it Mr. and Mrs. Young and their children passed pleasantly the hours of the evening between dark and bed time. One piece of furniture was a clock, which occupied a place on the mantel. The clock has been in the Young family for years, and has never been known to stop.

On Saturday evening, when Mr. Young reached home he informed his wife that he felt sick. He said that his head, his arms, his chest, his feet and his hands pained him intensely. He could not account for the feeling, and told his wife that he believed he was going to be sick. Mr. Young had always been strong and hearty. He had never complained of a pain or an ache and his wife at once had a good fire built in the bed-room and made her husband as comfortable as possible. His feet were bathed and every possible effort to alleviate his pains was made. Early in the evening Mr. Young retired, and soon after Mrs. Young sought her place in the same bed. Mr. Young's mouth and throat were parched, and frequent resorts to the water-pitcher was the result. Several times after retiring, Mrs. Young got up to give her husband water. Mr. Young was quite restless. He could not sleep. His wife was uneasy about him and watched his condition, noting every change with great anxiety. About midnight she got up to give Mr. Young water for the last time. As she handed him the water he said:

"The fire has gone out and you will take cold this way. Put the pitcher here by the bed and lie down and go to sleep. I will be all right in the morning."

Mrs. Young placed the pitcher near the bed and then walked up to the mantel and looked at the clock. It was then fifteen minutes after twelve. She then sought her bed again. Soon after lying down she observed that her husband was very quiet, and thinking he was asleep she turned over and, closing her eyes, was soon lost to the troubles of this world in a pleasant, dreamless sleep. Her sleep lasted till daylight, when she awoke.

Turning over she then saw that her husband's eyes were closed, and thinking that he was sleeping quietly and peacefully, she arose without making any noise and made the fire—her husband's usual task. Her heart would not let her disturb him to make the fire. He was sick and needed the rest. With these and other loving feelings in her heart for the man on the bed, she went on with her breakfast, little dreaming that she was cooking for the dead. While preparing breakfast Mrs. Young looked up at the clock. The hands pointed to two o'clock. This indicated that the clock was not running—an unheard of occurrence for that time-piece. Mrs. Young stepped up to the mantel and was astonished to find the clock still. She could not account for it, but soon dismissed the subject from her mind by thinking of her husband and the pleasant hours she would pass with him that day as he would not have to work. When breakfast was ready Mrs. Young called her husband gently, but receiving no response stepped up to the bed, and, laying her hand on him, was in the act of calling him again, when she suddenly threw up both hands, and falling across the bed, wailed: "My husband, oh, my husband, are you dead?" He was cold in death. The faint touch when Mrs. Young's hands came in contact with the icy body told her loving heart that the man she loved was dead. And it was true. He was dead, cold and stiff in death, but no one knows when he died. He was alive when Mrs. Young gave him the water at midnight, but he was dead when she called him for breakfast. He may have died at any moment. Death may have come in when the clock stopped. Stranger things have occurred.

Mr. Young was a well to do, hard working, deserving mechanic. He was about forty-five years of age. His death was sudden and Coroner Haynes decided that an inquest was necessary. A jury was organized, and after hearing the evidence a verdict of death from dropsy of the heart was returned.

